# Supplementary material for: Piezo1 mutant zebrafish as a model of idiopathic scoliosis
Source: Front Genet. 2024 Jan 8;14:1321379. doi: 10.3389/fgene.2023.1321379 (PMC10801085; doi:10.3389/fgene.2023.1321379)
Supplement: Supplementary file 1 [file DataSheet1.pdf]

Supplementary Material

1 Supplementary Tables

Table S1: Oligo sequences used for CRISPR-Cas9 gRNA generation

**Gene**	**Primers name**	**Sequences**
piezo1	piezo1 sgRNA exon 5	GCT AAT ACG ACT CAC TAT AGG GAG CCA CAG CAC ACC CTG GTT TTA GAG CT
piezo2a	piezo2a sgRNA Exon 4	GCT AAT ACG ACT CAC TAT AGG GCC ACG CTC ATC CGC CTC GTT TTA GAG CT
piezo1 in-frame mutant (11aa del)	piezo1-sg1F	TAG GAG CGA AAT ATG CAG GCT G
	piezo1-sg1R	AAA CCA GCC TGC ATA TTT GCG T
mstnb	mstnb-sg1-F	TAG GAG CCT TCC ACA GCC ACG G
	mstnb-sg1-R	AAA CCC GTG GCT GTG GAA GGC T

Table S2: Primers used for genotyping

**Gene**	**Primers**	**Sequences**
piezo1	piezo1 int-4F	TCC TGG GAC GTA ACA AAG CA
	piezo1 int-5R	AGG CCC AGA CTA ACA GCA TT
piezo2a	piezo2a int-4F	TTT GAC AAC AAA ATG ACT CAC TAA TTG
	piezo2a int-5R	CGA TGT ACA AAA AGC CAC CA
piezo1 in-frame mutant (11aa del)	piezo1 int-1F	AAA ATC ACA GCA GGG TGA AT
	piezo1 int-2R	GGC AGA CTA TTG CAA CAT TGA
mstnb	mstnb-ex1F	ACA TCC TTT AGC ACG CCT TG
	mstnb-int1R	CTG CGT AAA GGG TCT CTC CA

Table S3: Primers used for qPCR

Gene	Primers	Sequences
piezo1	piezo1 QPF	GAG AGG ATG CGG CTT CTC AA
	piezo1 QPR	CCA CAT GGT GAA TCC GTC CA
piezo2a	piezo2a QPF	CCG GAT AAC TAC ACC GAG GC
	piezo2a QPR	CCA GCA GGG GAC TGA ACT TT
osterix	osterix QPF	GAC CCT CAC TGG ACT GCT TC
	osterix QPR	CGA ATT TGT TGC AGG TCG CA
bglap	bglap QPF	CAG TCC TGA TCT TCT GCT GCC
	bglap QPR	CAC GCT TCA CAA ACA CAC CTT CAT

2 Supplementary Figures

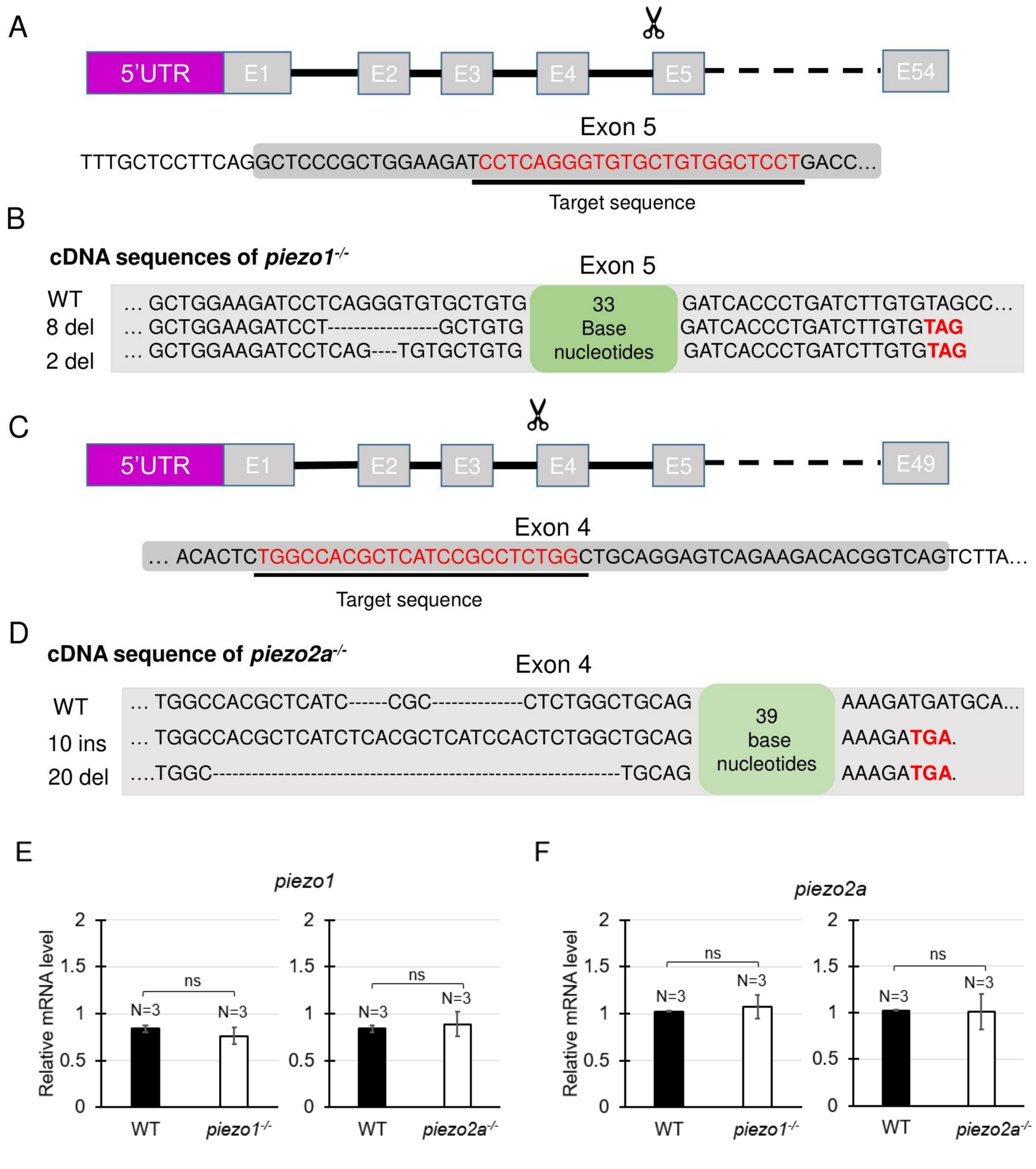

Supplementary Figure S1. Generation of *piezo1*$^{-/-}$ and *piezo2a*$^{-/-}$**.** (A)(C) Schematic diagram of CRISPR/Cas9 targeting of *piezo1* and *piezo2a* genes by guide RNAs targeting the N-terminal regions of *piezo1* exon 5 and *piezo2a* exon 4, respectively. (B)(D) Sequence confirmation of *piezo1*$^{-/-}$ alleles and *piezo2a*$^{-/-}$ alleles showing premature terminator codon. Relative mRNA level of (E) *piezo1* and (F) *piezo2a* in *piezo1*$^{-/-}$ and *piezo2a*$^{-/-}$ mutant fish. No significant changes in the expression of the

corresponding genes suggest that transcriptional adaptation and Non-mediated mRNA decay (NMD pathway are not required for genetic compensation in each piezo mutant.

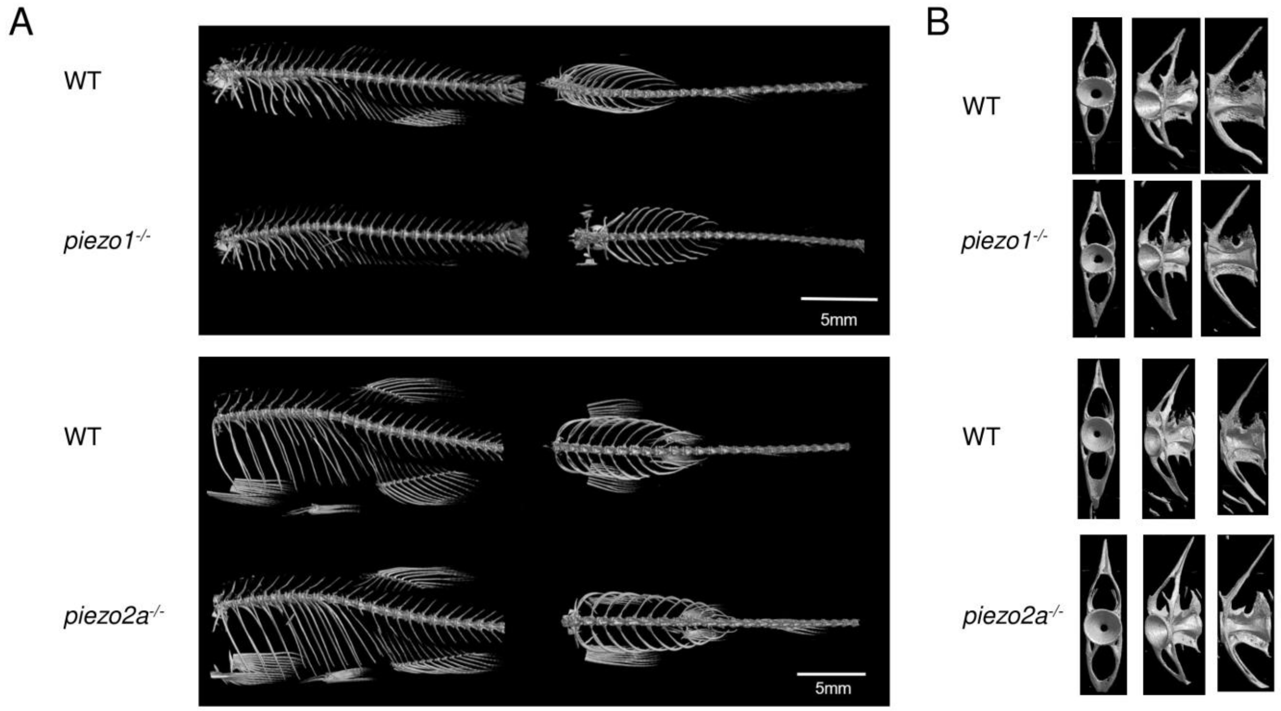

Supplementary Figure S2. Bone phenotypes of *piezo1*$^{-/-}$ and *piezo2a*$^{-/-}$. Comparison of 3D reconstruction of micro-CT images of (A) whole body and (B) bone segment between wildtype and *piezo1*$^{-/-}$ at 4 mpf (N=6) also wildtype and *piezo2a*$^{-/-}$ at 6 mpf (N=4). Notably, there were no morphological abnormalities in each null mutant.

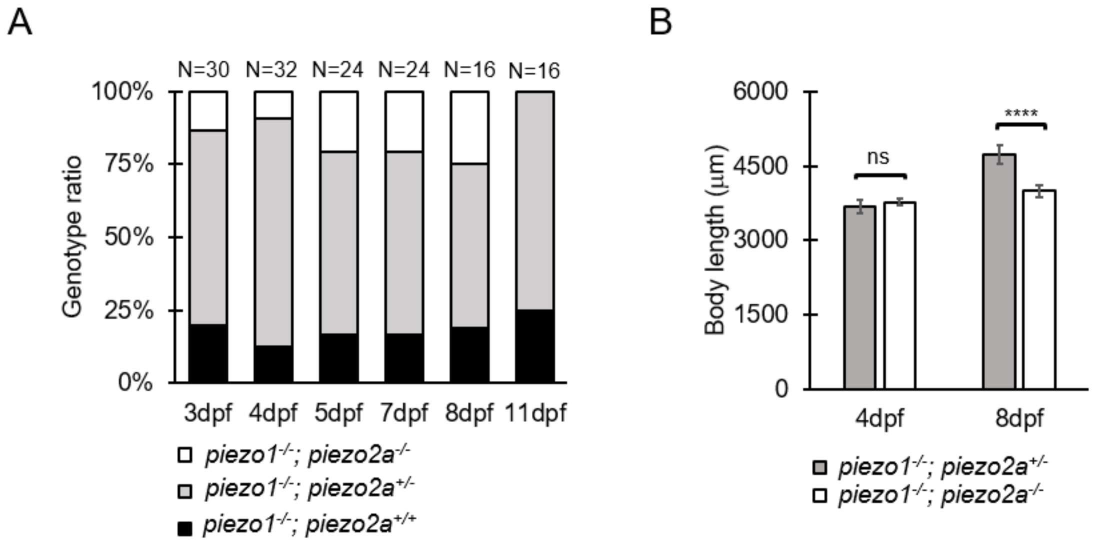

Supplementary Figure S3. Survival rate and total body length of *piezo1*$^{-/-}$; *piezo2a*$^{-/-}$. (A) Genotype ratio between double knock out and sibling in several time points. (B) Graph depicting total body length at 4dpf and 8dpf (N=8 for each group). Values are presented as mean ± SD and analyzed using student t-test. **** $P < 0.0001$. ns indicates not significant.

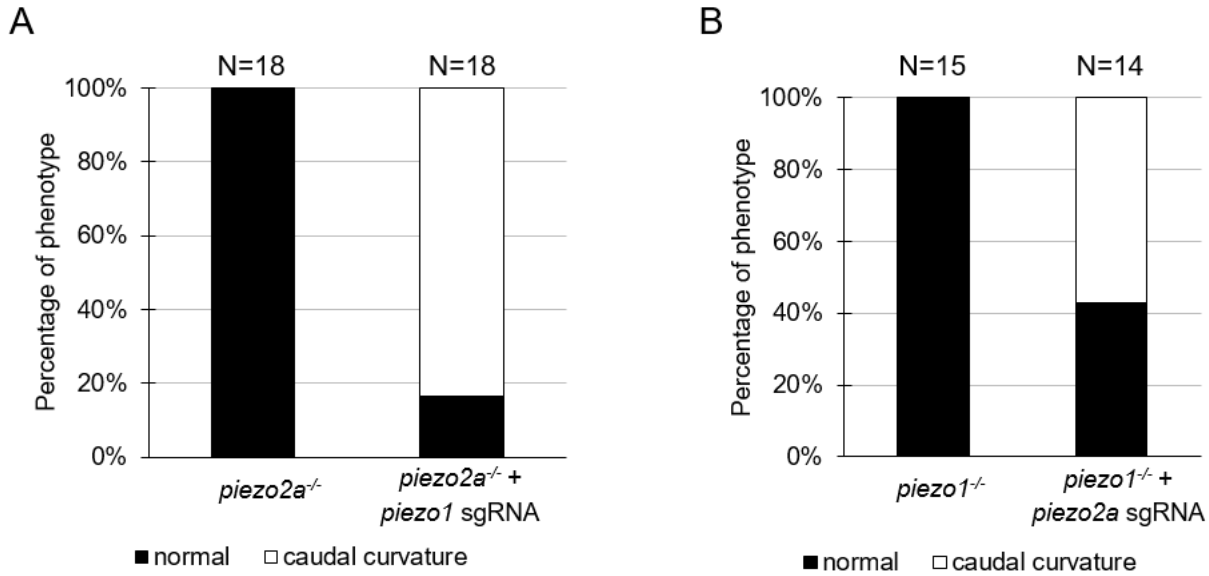

Supplementary Figure S4. Percentages of abnormal phenotype in mosaic mutant fish.

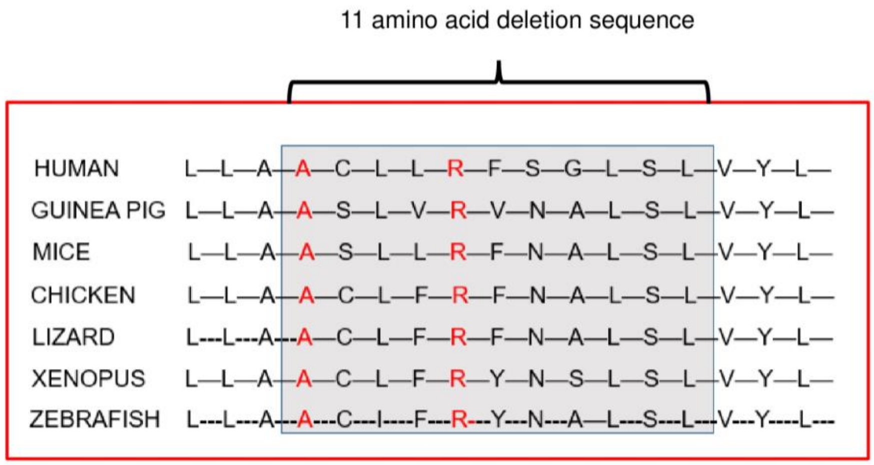

Supplementary Figure S5. 11 amino acid deletion region in Piezo1 is highly conserved among vertebrates.

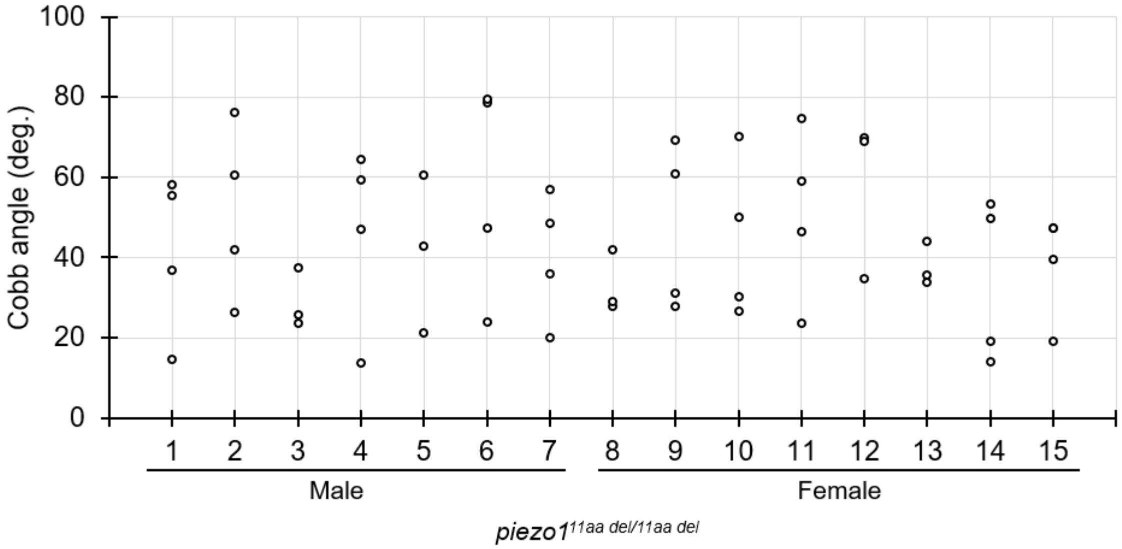

Supplementary Figure S6. Cobb angle measurement in scoliosis mutant fish according to sex. Circles represent angles for individual curves (Bearce et al., 2022).

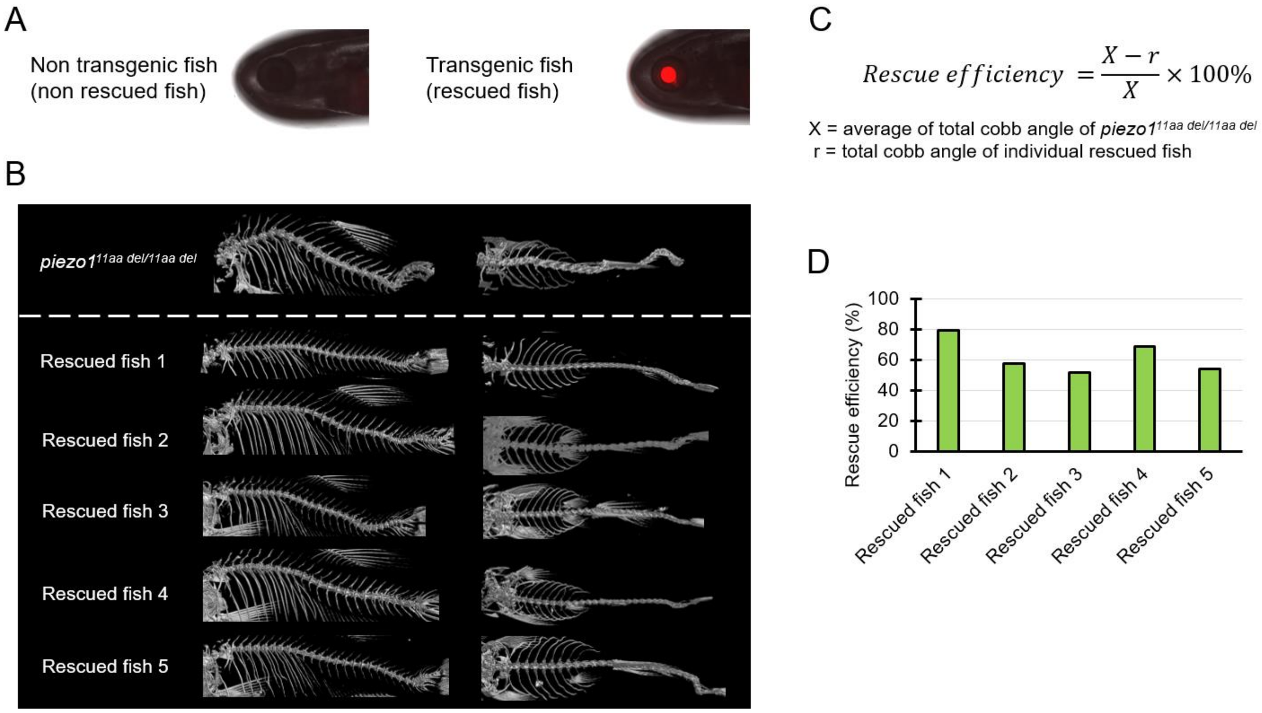

Supplementary Figure S7. Validation of rescue experiment. (A) Confirmation of successful integration of rescue plasmid in zebrafish genome. Rescue plasmid has *crystallin-pro:mCherry*. Once the plasmid is expressed, red fluorescent will be emitted in eyes, indicating a transgenic fish. (B) Phenotype variation of rescued mutant fish. (C) Formula of measuring rescue efficiency. Introduction rescue plasmid in *piezo1*$^{11aa\ del/11aa\ del}$ alleviates scoliosis symptoms, but (D) the efficiency is varied among the rescue fish.

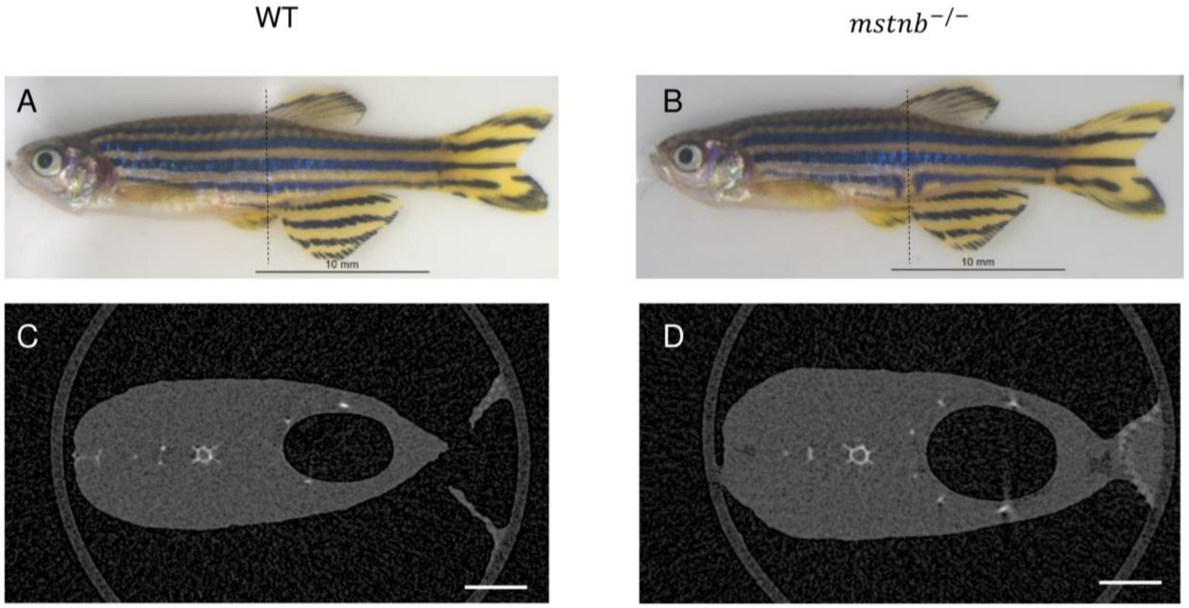

Supplementary Figure S8. Phenotype of *mstnb*$^{-/-}$. (A)(B) Gross phenotype between wildtype and *mstnb*$^{-/-}$ at 4 months after fertilization. Notably, the body size of *mstnb*$^{-/-}$ was slightly larger. (C)(D) Cross-sections view of abdominal part. The used scale is 50 μm.
